# Circulating Cell‐Free RNA Reflects Immune‐Associated and Airway Remodeling Signatures in Equine Asthma

**DOI:** 10.1155/jimr/5310284

**Published:** 2026-08-26

**Authors:** Birong Zhang, Hanna Agdour, Miia Riihimäki, Sanni Hansen, Amanda Raine

**Affiliations:** ^1^ Department of Medical Sciences, Uppsala University, Uppsala, Sweden, uu.se; ^2^ Science for Life Laboratory, Uppsala, Sweden, scilifelab.se; ^3^ Department of Clinical Sciences, Swedish University of Agricultural Sciences, Uppsala, Sweden, slu.se; ^4^ Department of Veterinary Clinical Sciences, University of Copenhagen, Copenhagen, Denmark, ku.dk

**Keywords:** airway disease, asthma, biomarker discovery, equine asthma, IL33, plasma cfRNA, transcriptomics

## Abstract

Circulating cell‐free RNA (cfRNA) has emerged as a promising minimally invasive biomarker capable of capturing transcripts originating from multiple tissues and cell types, but its utility in chronic inflammatory airway diseases remains poorly understood. Equine asthma (EA), a naturally occurring respiratory disease that shares key clinical and immunopathological features with human asthma, provides a valuable comparative model for evaluating plasma cfRNA in this context. We performed transcriptomic profiling of plasma cfRNA from horses with EA and healthy controls and compared these findings with partially matched bronchoalveolar lavage (BAL) and whole‐blood (WB) transcriptomes. Differential abundance analysis of plasma cfRNA identified increased abundance of the epithelial alarmin *IL33*, together with signatures associated with tissue remodeling, cellular stress responses, and immune alterations. Notably, cfRNA from EA horses exhibited reduced B‐cell–associated and increased T‐cell–associated transcript signatures, a pattern independently supported by differential abundance, co‐expression network, and deconvolution analyses. In contrast, similar immune‐associated signatures were not evident in the BAL and WB transcriptomes analyzed in this study. Cross‐compartment comparisons further demonstrated that plasma cfRNA captured a molecular profile largely distinct from those identified in BAL and WB, while sharing only a small set of differentially abundant transcripts across compartments. Collectively, these findings indicate that plasma cfRNA captures molecular patterns associated with EA that differ from those observed in conventional transcriptomic sampling compartments. These results support the potential utility of plasma cfRNA as a minimally invasive source of biomarkers for characterizing airway disease‐associated molecular and immune alterations in both veterinary and comparative asthma research.

## 1. Introduction

Equine asthma (EA) is a naturally occurring chronic inflammatory airway disease that shares clinical and molecular features with human asthma. Bronchoalveolar lavage (BAL) cytology remains the diagnostic gold standard in EA but provides limited insights into the molecular and cellular processes underlying disease pathogenesis. Whole‐blood (WB) transcriptomics can provide complementary information but often lacks disease‐specific signals observed locally in BAL and airway tissue transcriptomes, highlighting the need for alternative minimally invasive molecular biomarkers [1].

Severe EA (sEA) is predominantly characterized by neutrophilic airway inflammation, whereas mild‐to‐moderate EA (mEA) encompasses a heterogeneous spectrum of inflammatory phenotypes, including neutrophilic, mastocytic, eosinophilic, mixed, and paucigranulocytic forms [2, 3]. In addition to inflammation, airway remodeling processes involving epithelial barrier dysfunction, smooth muscle hyperplasia, and subepithelial fibrosis contribute to disease pathogenesis and have been described in both sEA and mEA [4–6]. The heterogeneous nature of EA, together with the invasiveness of current diagnostic approaches, presents challenges for disease and therapeutic monitoring and underscores the need for accessible biomarkers that capture both local and systemic disease processes.

Plasma cell‐free RNA (cfRNA) has recently emerged as a promising biomarker platform in oncology, prenatal medicine, and systemic inflammatory diseases [7, 8]. Released through cellular apoptosis, necrosis, and active secretion, circulating cfRNA may reflect gene expression patterns and ongoing biological processes across multiple tissues [9]. In plasma, cfRNA exists in multiple forms, including protein‐bound complexes, extracellular vesicles (EVs), and freely circulating RNA fragments [10, 11]. EV‐associated RNA cargo has attracted increasing interest in human asthma and related airway diseases, where altered EV RNA profiles have been associated with inflammatory phenotypes and disease activity [12–14].

Owing to its accessibility through routine blood sampling and its ability to capture transcripts released from multiple cell and tissue types, cfRNA provides a minimally invasive window into systemic molecular processes [15–18]. To date, plasma cfRNA has not been characterized in horses. In this exploratory study, we performed the first transcriptomic characterization of equine plasma cfRNA and compared plasma cfRNA profiles with partially matched BAL and WB transcriptomes. The aims of the study were to (i) identify differentially abundant transcripts and enriched biological pathways associated with EA, (ii) investigate putative cellular and tissue contributors to the circulating cfRNA pool in healthy horses and horses with EA, and (iii) explore potential shared and distinct molecular signatures across cfRNA, BAL, and WB transcriptomes.

## 2. Methods

### 2.1. Horses and Sampling Procedures

Horses diagnosed with EA were sampled during clinical evaluation at an equine referral hospital (University Animal Hospital, SLU, Uppsala, Sweden). EA diagnosis was based on clinical history, physical examination, and BAL cytology (Supporting Information 1: Table S1).

Inclusion criteria for EA cases were as follows: (i) a history of coughing, exercise intolerance, and/or unexplained hyperpnea during exercise for >3 weeks and (ii) BAL cytology showing ≥10% neutrophils and/or ≥3% mast cells. One horse with a history of coughing and exercise intolerance but normal BAL cytology was included in the EA group and classified as paucigranulocytic.

Healthy control horses (Standardbreds) were sampled during spring (April) at a national education and harness racing facility (Wången). Inclusion criteria for controls were as follows: (i) no history or current signs of coughing, exercise intolerance, or unexplained hyperpnea during exercise; (ii) unremarkable endoscopic examination findings; and (iii) normal BAL cytology counts (<8% neutrophils and <3% mast cells). One control horse had a BAL mast cell count of 3%, corresponding to the diagnostic threshold for mastocytic EA, but was retained in the control group based on an unremarkable clinical history and endoscopic findings consistent with a healthy airway.

On average, EA horses were older than controls (cfRNA: *p* = 0.006; BAL: *p* = 0.002; WB: *p* = 0.060, Welch’s *t*‐test), whereas sex distribution did not differ significantly between groups (Supporting Information 1: Table S1 and Supporting Information 2: Table S2). Blood parameters were not evaluated in this cohort. Neither EA cases nor control horses exhibited clinical signs of infection or had any known comorbidities at the time of sampling.

BAL and BAL cytology were performed as previously described [19]. WB and plasma were collected on the same sampling occasion as BAL from the jugular vein using Tempus Blood RNA tubes (Thermo Fisher) and Streck RNA Complete BCT tubes (Streck). The Tempus tubes contain reagents that lyse and stabilize blood cells immediately upon collection, preserving the intracellular RNA. Filled tubes were stored at −80°C until processing. Streck RNA Complete BCT tubes contain stabilizing agents that preserve the integrity of nucleated blood cells and prevent their lysis, as well as maintain the quality of cfRNA in plasma. Plasma was extracted from the Streck tubes according to the manufacturers instruction and stored at −80°C until processing. Ethical permissions (#5.2.18‐4819/15 and #31‐2605/04) were granted by the regional ethical board.

### 2.2. RNA Extraction

Total RNA was extracted from BAL cells using the Qiagen AllPrep Kit. WB RNA was isolated from Tempus tubes using Norgen’s Total RNA Purification Kit. cfRNA was extracted from 2 mL of plasma using the Quick‐cfRNA Serum and Plasma Kit (Zymo Research) without prior enrichment for EVs. RNA integrity was checked on a TapeStation system (Agilent) using RNA Screen Tapes (BAL and WB, RIN > 8) or High Sensitivity RNA Screen Tapes (cfRNA, RIN ≤ 3). cfRNA yields were, as expected, very low (1–5 ng total RNA/mL plasma) and dominated by short molecules. cfRNA yields were generally too low to be reliably quantified by TapeStation or Qubit analysis, so the eluted sample volume was reduced by vacuum centrifugation, and the entire concentrate was used for library preparation. All RNA samples, including cfRNA, were DNase I‐treated just prior to library prep using the Heat&Run gDNA removal kit (ArcticZymes Technologies).

### 2.3. RNA‐Seq Library Preparation

RNA‐seq libraries were prepared using the SMARTer Stranded Total RNA‐Seq v3—Pico Input Mammalian Kit (including unique molecular identifiers [UMIs]) or the v4 version, renamed SMART‐Seq Total RNA Pico Input with UMIs (ZapR Mammalian), which according to the manufacturer (Takara Bio) is the same kit as v3 but exhibits improved rRNA removal efficiency. The fragmentation step was omitted for the cfRNA samples. A total of 10 ng of RNA input was used for BAL and WB samples (maximum input specified for this kit). For cfRNA, the entire amount extracted from 2 mL of plasma was used as the input (estimated at ~1–5 ng based on TapeStation profiles). Libraries were sequenced as paired‐end 150 bp (PE150) reads on a NovaSeqX instrument. The reason for switching to the new version of the kit (v4) was that the removal of equine ribosomal RNA proved rather inefficient with the v3 kit. However, we did not observe a significant improvement in rRNA removal efficiency with the v4 kit. Principal component analysis (PCA) indicated that batch effects across the v3 and v4 library prep kits were insignificant.

### 2.4. RNA‐Seq Read Processing

Best practice alignment, UMI deduplication, and quantification were performed with the nf‐core/RNA‐seq pipeline v.3.8.1 (https://github.com/nf-core/rnaseq), using EquCab 3.0 annotation version 0.111. The number of unique mapped reads (STAR v.7.10a) was on average 16.8 ± 9.1 million reads for the cfRNA samples, 33.9 ± 8.8 for the WB samples and 33.7 ± 16.6 for BAL samples. UMI deduplication was performed with UMI‐tools within the nf‐core RNA‐seq pipeline (v.1.1.2). Ribosomal reads were higher than expected (Subread_featurecounts v2.0.1), considering the library kit is designed for mammalian rRNA removal: average 39.9% in cfRNA, 32.7% in WB and 29.9% in BAL‐data. (Supporting Information 3: Data 1). After excluding ribosomal genes, 38,666 of the initial 38,849 annotated genes were retained for downstream analysis across all three compartments. No normalization for estimated blood volume was performed, as both tissue mass and blood volume generally scale with body size, whereas the relationship between this scaling and circulating cfRNA abundance per unit plasma volume has not been established.

### 2.5. Differential Gene Abundance and Pathway Analysis

Differential gene abundance/expression analysis was conducted independently for each experimental dataset using the DESeq2 R package (v.1.48.1) [20]. For the plasma cfRNA compartment, the term differential abundance analysis was used to distinguish it from conventional tissue differential gene expression (DGE) analysis. Changes in cfRNA abundance may reflect a complex interplay of RNA release, clearance, tissue contributions, cell turnover, EV dynamics, and cellular composition and therefore do not necessarily reflect transcriptional regulation in the tissues from which the RNA originated. In contrast, for the cellularly derived samples (BAL and WB), we use the established term DGE. Age was included as a covariate in the cfRNA dataset (Supporting Information 2: Table S2) but not in the BAL dataset due to the small sample size and heterogeneity of the EA group, which could lead to overfitting and reduce statistical power. WB dataset did not differ significantly by age and were therefore analyzed without age adjustment. Breed was not included as a covariate in the statistical model because its effects are not separable from disease status (controls were standardbreds and no case was standardbred). Significance criteria were as follows: Benjamini–Hochberg adjusted *p*‐value <0.05 and absolute log_2_ fold change (|log_2_FC|) > 1.0.

Functional enrichment analyses were performed using clusterProfiler (v4.16.0) against the KEGG database. Over‐representation analysis (ORA) was applied to significant genes, while gene set enrichment analysis (GSEA) was performed on genes ranked by log_2_ fold change [21]. Pathways with an adjusted *p*‐value <0.05 were considered significantly enriched.

### 2.6. Deconvolution Analysis

Cell deconvolution analysis was performed on equine bulk RNA sequencing data to investigate relative tissue contributions. Cross‐species ortholog mapping was performed using the biomaRt R package (v2.64.0) [22]. To ensure high‐confidence cross‐species comparisons, only orthologous relationships with a confidence score of 1 were retained, which successfully mapped 15,800 equine genes to their human orthologs.

For cfRNA, cell signature fractions were estimated via CIBERSORTx, following the procedure established by Vorperian et al. [23], using two publicly available reference matrices: the human tsp_v1_basisMatrix (Tabula Sapiens, TSP) and the LM22 immune cell reference [24]. Both reference and bulk RNA‐seq data were normalized to counts per million (CPM) prior to CIBERSORTx input, as the human tsp_v1_basisMatrix was originally derived from CPM‐normalized expression value and as CIBERSORTx requires consistent normalization between the reference matrix and the mixture data. To ensure methodological robustness, three independent methods were applied for cross‐validation. Weighted least squares (WLS) and dtangle [25], like CIBERSORTx, estimate relative cell‐type proportions and were run with both references, whereas xCell uses its own built‐in gene signatures to compute enrichment scores rather than proportions [26].

For BAL and WB bulk RNA‐seq data, deconvolution was performed using CIBERSORTx. Cell fractions were estimated using equine single‐cell reference datasets (BAL: PRJNA914226 [19] and PBMC: GSE148416 [27]) and the LM22 reference matrix.

### 2.7. Weighted Gene Co‐Expression Network Analysis

WGCNA was performed on cfRNA‐seq data to identify co‐expression modules associated with disease status [28]. Raw count data were filtered to retain genes with counts >1 in at least three samples, followed by the removal of genes with variance below the 25th percentile to eliminate low‐information features. The filtered count matrix was then normalized using DESeq2’s variance‐stabilizing transformation (VST). To focus on the most biologically informative and variable genes while maintaining computational tractability, genes with variance above the 95th percentile were selected for network construction, yielding approximately 1000 genes. WGCNA was performed using a soft‐thresholding power of 12, determined by the scale‐free topology criterion, resulting in the identification of six distinct co‐expression modules. Module–trait correlation analysis was conducted to assess the relationship between each module and asthma status/age. Module_ turquoise exhibited significant negative correlation with asthma, indicating that genes within this module were predominantly downregulated in asthmatic horses relative to controls, while Module_ blue showed significant positive correlation with asthma, suggesting upregulation of its constituent genes in diseased animals. Genes from these two disease‐associated modules were extracted for subsequent expression pattern visualization and KEGG pathway over‐representation.

### 2.8. Comparative Transcriptomic Profiling

To evaluate transcriptomic concordance and divergence across cfRNA, BAL, and WB compartments, integrative comparative analyses were performed. PCA was conducted using transcript‐level expression values quantified at transcripts per million (TPM) for all detected genes to visualize global expression patterns and assess sample clustering across compartments. Stat values—DESeq2‐derived Wald statistics reflecting both the direction and significance of gene expression changes—were extracted from independent differential expression analyses of each compartment. The distributions of statistical values were compared, and pairwise Spearman correlations were calculated to characterize their respective transcriptional response profiles. Additionally, the overlap of DEGs (adjusted *p*‐value <0.05 and |log_2_FC| > 1) across the three compartments was calculated to identify shared and compartment‐specific transcriptional signatures associated with EA.

### 2.9. Statistical Analysis

All statistical analyses were conducted using R version 4.5.0 (https://www.r-project.org). Prior to statistical testing, data distributions were assessed using the Shapiro–Wilk test. Normally distributed data were analyzed using Welch’s *t*‐test for two‐group comparisons and Pearson correlation (reported as *r*), while non‐normally distributed data were analyzed using the Wilcoxon rank‐sum test (Mann–Whitney *U* test) and Spearman correlation (reported as *ρ*). As the Wilcoxon rank‐sum test was the predominant statistical test applied throughout this study, it is not explicitly labeled; Welch’s *t*‐test is labeled where it is applied. Full details of normality testing, statistical tests, and correlation methods for each analysis are provided in Supporting Information 4: Data 2. When multiple comparisons were performed, *p*‐values were adjusted using the Benjamini–Hochberg procedure to control the false discovery rate. Statistical significance was defined as an adjusted *p*‐value <0.05.

## 3. Results

### 3.1. EA Samples Collection and Multiple Biological Compartments Study Design

**Figure 1:**
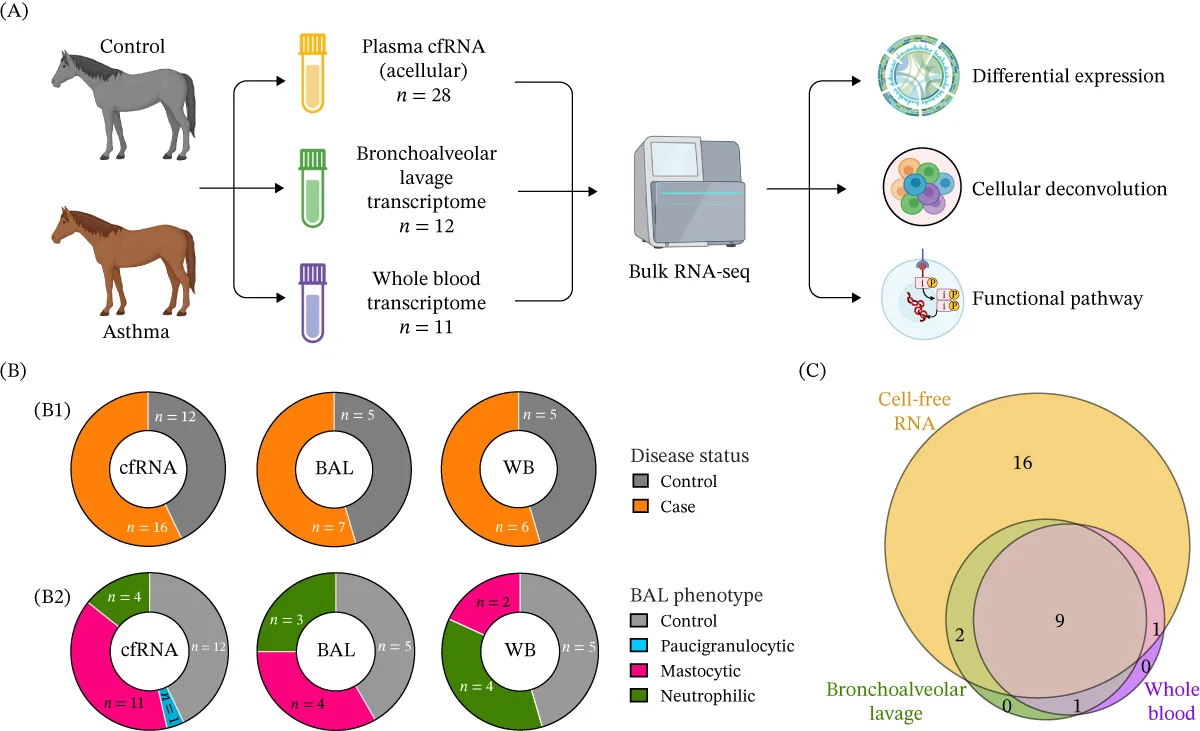
Sample distribution and overlap across biological compartments in equine asthma transcriptomics study. (A) Study design. Schematic overview of the multicompartment sampling strategy. (B) Sample size distribution by experimental groups. (B1) Distribution and numbers of samples for control (gray) and equine asthma (orange) groups across compartments. (B2) Distribution by BAL cytological phenotype within each compartment, stratified by control (light gray), paucigranulocytic (blue), mastocytic (pink), and neutrophilic (green) groups. (C) Sample overlap. Venn diagram illustrating the intersection of 29 samples collected across the three biological compartments. Illustrations created using BioRender.

We collected samples from three biological compartments in a clinically recruited cohort of horses with EA (*n* = 16) and healthy controls (*n* = 12) to comprehensively characterize the plasma cfRNA landscape and compare it with BAL and WB transcriptional signatures (Figure 1A). cfRNA‐stabilizing blood collection tubes were used to minimize ex vivo release of platelet‐ and leukocyte‐derived RNA, in combination with a total RNA‐seq workflow optimized for low‐input and incorporating UMIs to reduce amplification bias. The EA cases included diverse BAL cytology phenotypes, including paucigranulocytic (*n* = 1, mEA), mastocytic (*n* = 11, mEA), and neutrophilic (*n* = 4, sEA) (Figure 1B and Supporting Information 1: Table S1). For comparative analysis, we sequenced BAL RNA (*n* = 12; 7 EA cases, 5 controls) and WB RNA (*n* = 11; 6 EA cases, 5 controls) from partially matched individuals using the same library preparation protocol. (Figure 1A,B and Supporting Information 1: Table S1). These samples similarly represented both mastocytic and neutrophilic phenotypes (Figure 1B). We obtained full three‐compartment sampling from nine horses, with additional horses contributing two‐compartment pairs. (Figure 1C). The demographic characteristics of all study subjects are summarized in Supporting Information 1: Table S1 and Supporting Information 2: Table S2. EA cases were on average older than controls (cfRNA: *p* = 0.006; BAL: *p* = 0.002; WB: *p* = 0.06, Welch’s *t* test), whereas sex distribution did not differ significantly between groups (Supporting Information 2: Table S2).

At a threshold of ≥1 CPM, cfRNA samples yielded an average of 9988 detected genes per sample, compared with 10,379 and 10,577 detected genes in BAL and WB samples, respectively. The detected transcripts in plasma cfRNA spanned multiple biotypes: predominantly protein‐coding genes (56%) and long noncoding RNAs (39%), with smaller contributions from miRNA (1.2%), snRNA (1.0%), snoRNA (0.96%), and various pseudogene and immunoglobulin/T‐cell receptor gene segments (<1%) (Supporting Information 5: Table S3). The main focus of this study was protein‐coding RNA and other long cfRNA transcripts, rather than microRNAs (miRNAs), in plasma.

### 3.2. Plasma cfRNA in EA is Characterized by Tissue Remodeling, Cellular Stress, and Immune‐Associated Signatures

**Figure 2:**
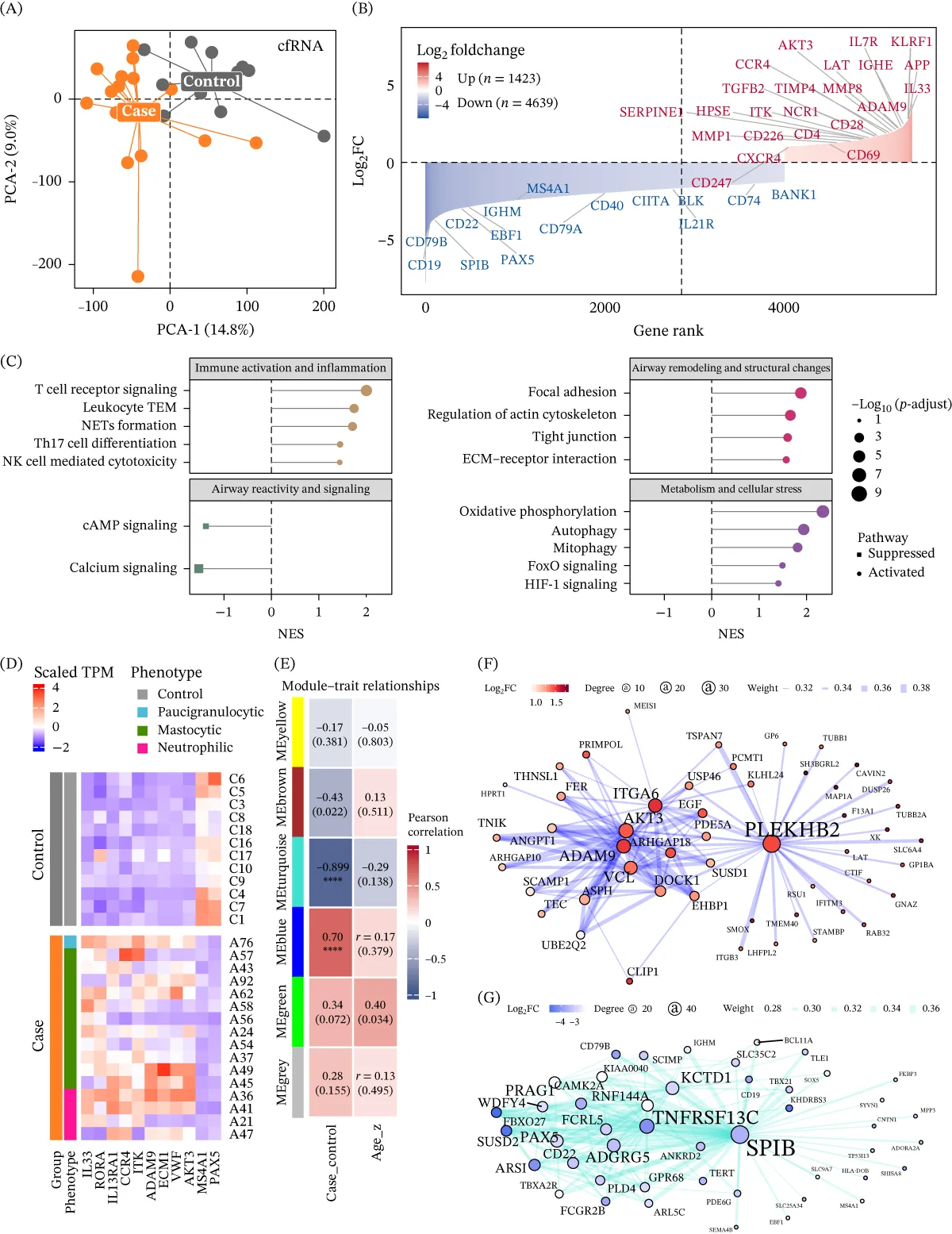
cfRNA profiling reveals disease‐associated transcriptional signatures in plasma samples from equine asthma cases. (A) Principal component analysis. PCA reveals distinct transcriptomic profiles in cases (*n* = 16) and controls (*n* = 12). (B) Ranked differential abundance plot. Transcripts with |log_2_FC| > 1 and *p*_adj_ <0.05 are shown (DESeq2). Selected genes are highlighted. (C) Pathway enrichment analysis. Lollipop plot showing GSEA results for significantly altered biological pathways (KEGG) between EA cases and controls. (D) Abundance patterns across asthmatic and control subjects. Heatmap of selected differentially abundant asthma‐associated genes (identified from human asthma literature). (E–G) Co‐expression network analysis. (E) Heatmap of correlations between WGCNA module eigengenes and disease status/age, with correlation coefficients and corresponding *p*‐values shown in each cell. Pearson correlations were used for associations with scaled age when data were normally distributed (ME_brown and ME_grey). WGCNA‐derived gene co‐expression networks for the blue (F) and turquoise (G) modules illustrating intramodular connectivity and gene‐correlation patterns. Nodes represent genes and edges represent co‐expression relationships. Node colors indicate log_2_ fold change (EA vs. control) from cfRNA differential abundance analysis, node size reflects connectivity (degree), and edge thickness represents co‐expression strength. Larger nodes denote highly connected hub genes.  ^∗∗∗∗^ *p* < 0.0001.

To characterize plasma cfRNA alterations associated with EA, differential transcript abundance analysis was performed. PCA indicated separation between EA and control cfRNA profiles (Figure 2A). Analysis of the combined cohort identified the asthma‐associated alarmin *IL33* among the most strongly elevated transcripts in horses with EA (log_2_FC = 3.2, *p*  < 10^−4^) [29]. Increased abundance of *IL13RA1*, involved in IL‐13/IL‐4 receptor signaling (log_2_FC = 1.8, *p*  < 10^−4^), the transcription factor *RORA* (log_2_FC = 1.3, *p*  < 10^−4^) and *IGHE* (log_2_FC = 2.42, *p* ≤ 10^−3^), further supported altered immune‐associated cfRNA signatures in EA. Notably, these genes have previously been implicated in asthma and allergic airway disease in humans, although their precise roles in EA remain incompletely understood [29–31].

Additional transcripts elevated in EA horses included extracellular matrix (ECM) and remodeling mediators (e.g., *ECM1*, *VWF*, *MMP1*, *MMP8*, *SERPINE1*, *ADAM9*, *HPSE*, *TGFB2*, *SDCBP*, *TIMP3*, and *TIMP4*), the kinase signaling component (*AKT3*), the cytoskeletal regulator (*KRT16*) and platelet‐associated factors (e.g., *APP*) [29, 32–39]. Lymphocyte‐associated transcripts were also prominent, including genes involved in T‐cell activation and signaling (*CD3D*, *CD28*, *CD4*, *CD226*, *CD247*, *LAT*, *ITK*, *CD69*, and *IL7R*), cytotoxic‐T/NK‐cell‐associated molecules (*KLRF1*, *NCR1*, *GZMK*, and *GZMH*), and chemokine receptors (*CCR4* and *CXCR4*) (Figure 2B,D). A complete list of differentially abundant transcripts is provided in Supporting Information 6: Data 3.

By contrast, B‐cell developmental and functional transcripts were underrepresented in plasma cfRNA samples from EA horses. These included canonical B‐cell markers (*MS4A1*, *CD19*, *CD79A/B*, and *CD22*), lineage regulators (*PAX5*, *EBF1*, and *SPIB*), proximal signaling components (*BANK1* and *BLK*), selected Fc receptor family members, and antigen‐presentation/co‐stimulatory molecules (*CIITA*, *DMB*, *CD74*, and *CD40*) [40, 41]. Concordantly, multiple immunoglobulin locus transcripts (*IGHM*, *IGHV*, and *IGLC3*) were reduced (Figure 2B).

KEGG pathway analysis revealed activation of immune activation pathways, including T‐cell receptor signaling, leukocyte migration, neutrophil extracellular trap (NET) formation, and, to a lesser extent, NK cell‐mediated cytotoxicity and Th17 differentiation. Alongside these, several pathways related to cell adhesion, ECM interactions, and structural organization were enriched, including focal adhesion, ECM–receptor interaction, regulation of the actin cytoskeleton, and tight junction signaling. Collectively, these findings were consistent with plasma cfRNA signatures associated with tissue remodeling and barrier‐related processes. Moreover, oxidative phosphorylation was strongly enriched and accompanied by increased autophagy, mitophagy, HIF‐1, and FoxO signaling, indicating coordinated signatures of metabolic and cellular stress‐response (Figure 2C).

Several KEGG pathways annotated as neurodegenerative diseases were also significantly activated, likely reflecting shared mitochondrial and oxidative stress‐related components within these broadly defined pathway annotations rather than neuro‐specific degenerative pathology (Supporting Information 7: Table S4). Calcium and cAMP signaling were among the few negatively enriched pathways in EA cases.

To determine whether the transcript‐level alterations identified by differential abundance analysis reflected coordinated biological programs rather than isolated gene‐level changes, we performed WGCNA on the cfRNA dataset. WGCNA was used to identify co‐expressed transcript modules associated with the EA status. Module–trait analysis revealed that gene co‐expression modules were predominantly associated with EA status rather than age (Figure 2E). The most significant co‐upregulated module (ME_blue, *n* = 184 genes) was positively correlated with disease status (*ρ* = 0.70, *p*  < 10^−4^) and was enriched for pathways related to oxidative phosphorylation, ECM–receptor interaction, regulation of the actin cytoskeleton, and platelet activation. (Supporting Information 8: Figure S1A,C and Supporting Information 9: Table S5). Within this module, hub genes, including *ADAM9*, *AKT3*, *VCL*, *DOCK1*, *ITGA6*, and *ARHGAP18*, exhibited high intramodular connectivity, indicating that transcripts associated with tissue remodeling formed coordinated co‐expression networks rather than isolated transcript‐level changes (Figure 2F).

The most significantly downregulated module (ME_turquoise, *n* = 215 genes, *ρ* = –0.899, *p*  < 10^−4^) was enriched for B‐cell‐associated genes, including canonical B‐cell markers (*MS4A1*, *CD19*, *CD22*, *CD79B*, *PAX5*, *EBF1*, *BCL11A*, and *SPIB*), B‐cell receptor signaling components (*FCRL5*, *SCIMP*, *SYVN1*, *FCGR2B*, and *TNFRS13C*), and immunoglobulin‐related transcripts (*IGHM*), providing independent network‐level support for the reduced B‐cell‐associated signatures identified by differential abundance analysis (Figure 2G, Supporting Information 8: Figure S1B,C and Supporting Information 9: Table S5).

PCA did not reveal significant group‐level separation of cfRNA transcriptomes among the different EA/BAL phenotypes included in this study (Supporting Information 10: Figure S2A). Given the pilot nature of this study and the need for increased statistical power, we limited our main analysis to the joint cohort. Nevertheless, DGE and GSEA results for mastocytic (*n* = 11) and neutrophilic (*n* = 4) cases versus controls are provided in Supporting Information 6: Data 3 and Supporting Information 10: Figure S2, including differential expression results for each comparison and the overlap of DEGs and enriched pathways.

### 3.3. Characterization of Putative Tissue Contributions to the Plasma cfRNA Composition in EA Horses and Controls

Deconvolution analysis is increasingly used to investigate the putative tissue and cellular origins of plasma cfRNA. To characterize potential contributors to the equine cfRNA pool, we performed deconvolution using a human pan‐tissue reference derived from TSP and the immune cell‐specific LM22 reference matrix following the orthology mapping of equine genes to human gene symbols. Deconvolution was performed using CIBERSORTx [24], a support vector regression–based method for estimating relative cellular contributions from bulk transcriptomic profiles. This approach is conceptually similar to recently established human cfRNA deconvolution frameworks that leverage single‐cell reference atlases and support vector regression to infer cell and tissue contributions to circulating cfRNA [23]. In the absence of equine pan‐tissue reference datasets, these analyses should, however, be considered hypothesis‐generating and interpreted as indicative of the tissue types that may contribute to the equine plasma cfRNA pool rather than as precise estimates of cellular composition or tissue‐of‐origin.

Application of this approach suggested that diverse cellular and tissue sources may contribute to the equine plasma cfRNA pool, with immune‐cell associated signatures comprising the predominant component, including B cells, T cells, myeloid and dendritic cells, NK/cytotoxic cells, platelets, and neutrophils, in line with findings from studies of human plasma cfRNA (Figure 3A). Signatures consistent with epithelial and stromal compartments were also detected, including intestinal epithelial cells, as well as endothelial, basal, muscle, and peripheral nervous system cell types. Notably, signatures suggestive of a potential contribution of respiratory epithelial cells to the circulating cfRNA pool were detected. (Figure 3A). Collectively, these findings suggest that equine plasma cfRNA captures transcripts originating from a broad range of immune and tissue compartments, supporting its potential utility as a minimally invasive source of systemic molecular information.

Figure 3Deconvolution analysis indicates differential cellular contribution to plasma cfRNA in equine asthma. (A) Comprehensive tissue‐of‐origin profiling across all cfRNA samples. Cross‐species cell signature deconvolution in combined cases and control samples, based on the Tabula Sapiens (TSP) pan‐tissue single cell reference data set. (B) Disease‐associated differences in cell‐type associated transcriptional signatures. Hierarchical clustering heatmap displaying *Z*‐score scaled TSP cell‐type signatures across equine asthma cases and controls. (C) Leukocyte deconvolution using LM22 reference. Stacked area plots illustrating the proportional distribution of 10 major immune cell populations estimated using the LM22 signature matrix. Sub cell‐types were collapsed into their major cell type (B‐cells, T cells, etc.). (D) Cross‐reference concordance of deconvolution methods. Forest plot showing correlations between cell fraction estimates generated using the TSP and LM22 reference matrices for seven overlapping immune cell populations. Error bars represent 95% confidence intervals. Statistical significance:  ^∗∗∗∗^ *p*  < 0.0001,  ^∗∗^ *p*  < 0.01; ns (not significant, *p*  > 0.05). (E) Replication of key immune signature alterations across deconvolution approaches. Dot plots comparing B cell and T cell signature fractions between control and case groups using two independent deconvolution approaches: TSP reference (E1) and LM22 reference (E2). Each dot represents an individual sample. Thick horizontal lines indicating group medians with interquartile range (IQRs; 25th–75th percentiles) ( ^∗∗∗∗^ *p*  < 0.0001). CSF, cell signature fraction.  ^∗^ *p* < 0.05,  ^∗∗∗^ *p* < 0.001.

**Figure:**
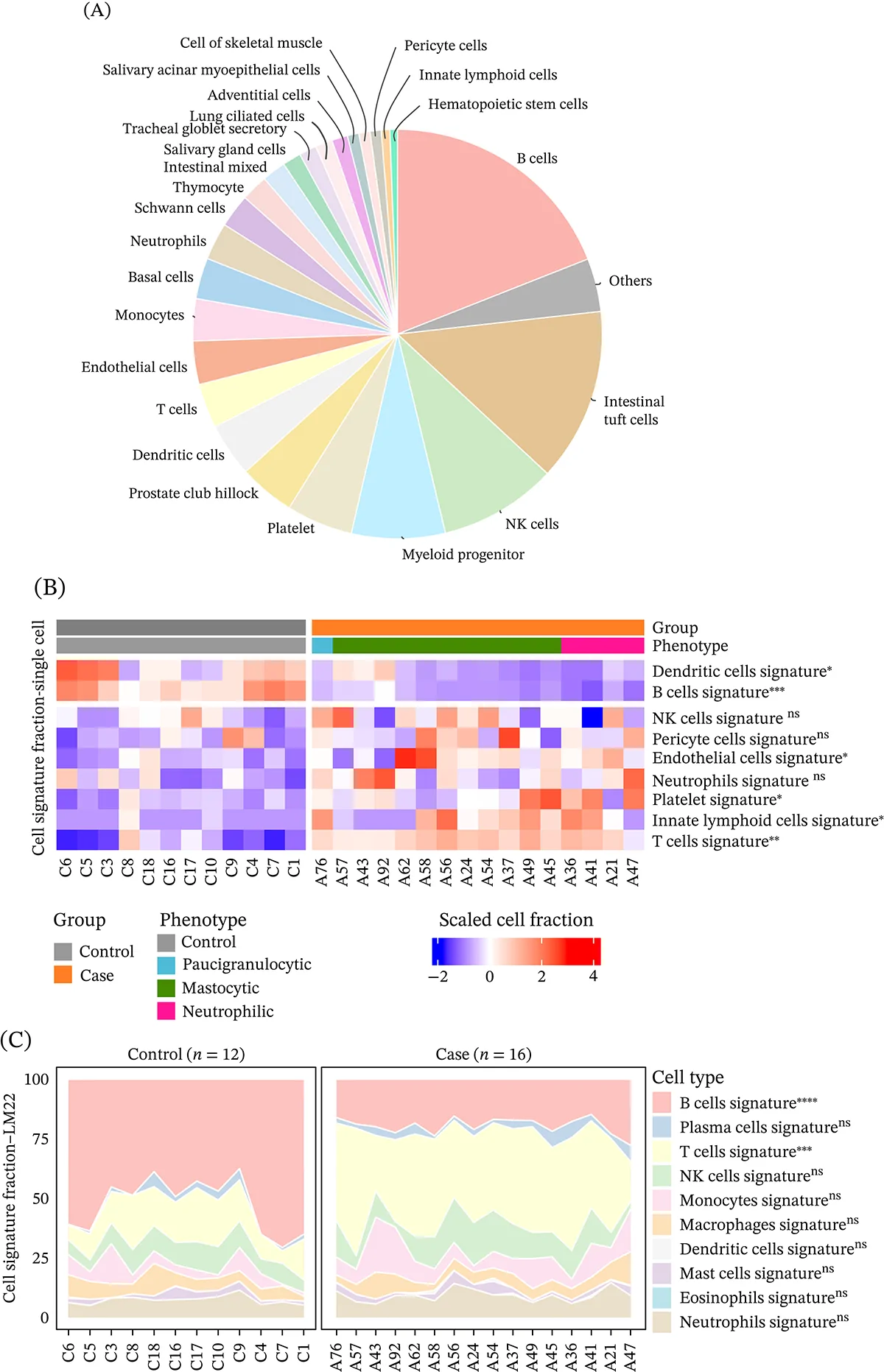

**Figure:**
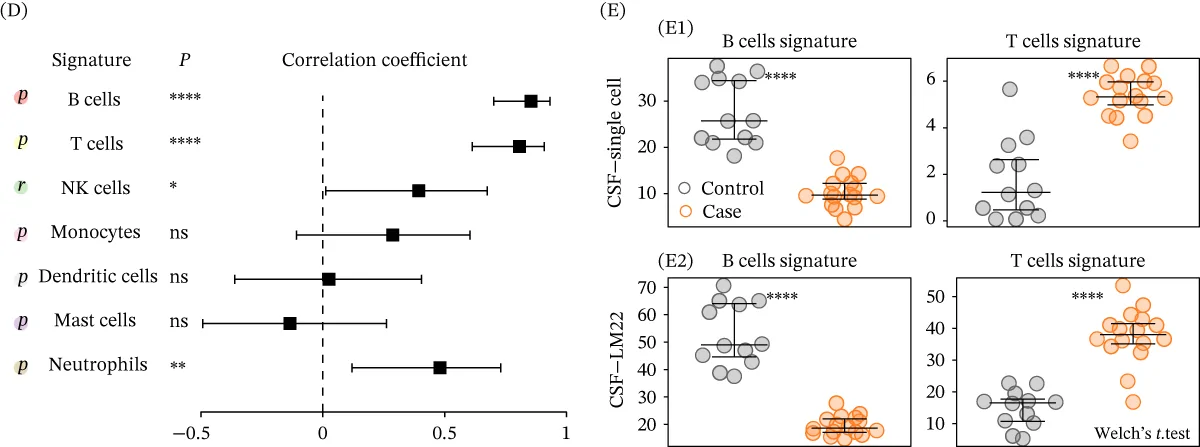

Comparative analysis of EA cases and healthy controls recapitulated the B‐cell‐ and T‐cell‐associated transcript signatures identified by differential abundance analysis. B‐cell‐associated signatures were reduced (TSP: *p*  < 10^−4^; LM22: *p*  < 10^−4^), whereas T‐cell‐associated signatures were increased (TSP: *p*  < 10^−4^; LM22: *p*  < 10^−4^) in EA samples. These findings were highly concordant between the two reference datasets for both B cells (*ρ* = 0.858, *p*  < 10^−4^) and T cells (*ρ* = 0.949, *p*  < 10^−4^) (Figure 3B–E). A more fine‐grained leukocyte‐focused deconvolution based on the human LM22 reference matrix is provided in Supporting Information 11: Figure S3. Interestingly, this analysis indicated reduced T‐follicular signatures concurrent with the reduction in B‐cell‐associated signatures in EA horses.

To further evaluate the robustness of these findings, B‐cell and T‐cell signatures were further assessed using three independent deconvolution methods (WLS and dtangle [25] using both single‐cell and LM22 references, together with the signature‐based enrichment method xCell [26]), all of which recapitulated the reduced B‐cell and increased T‐cell signatures observed in EA samples (Supporting Information 12: Figure S4).

Additionally, in agreement with the differential abundance analysis, platelet‐ (log_2_FC = 0.85, *p*_adj_ = 0.02), endothelial‐ (log_2_FC = 0.87, *p*_adj_ = 0.02), and innate lymphoid cell‐ (log_2_FC = 6.97, *p*_adj_ = 0.02) associated signatures were increased in EA samples (Figure 3B, Supporting Information 13: Table S6). NK‐cell (log_2_FC = 0.12, *p*_adj_ = 0.41) and neutrophil (log_2_FC = 0.6, *p* = 0.05, *p*_adj_ = 0.13) signatures (corresponding to the enrichment of NK cell‐mediated cytotoxicity and NET formation pathways) were not statistically significant but are nevertheless shown in Figure 3B. Dendritic cell (log_2_FC = −2.18, *p*_adj_ = 0.02) signatures were reduced in EA cases when using the TSP reference matrix; however, this pattern was not reproduced with LM22‐based deconvolution (Figure 3D).

Collectively, differential abundance analysis, co‐expression network analysis, and deconvolution converged on a common pattern characterized by reduced B‐cell‐associated and increased T‐cell‐associated cfRNA signatures in horses with EA. While these findings do not directly establish changes in cellular abundance or function, the concordance across multiple analytical approaches supports a robust shift in the immune‐related transcript composition of the plasma cfRNA pool in this EA cohort.

### 3.4. Transcriptional Patterns in BAL Cells and WB Across EA Cases and Controls

**Figure 4:**
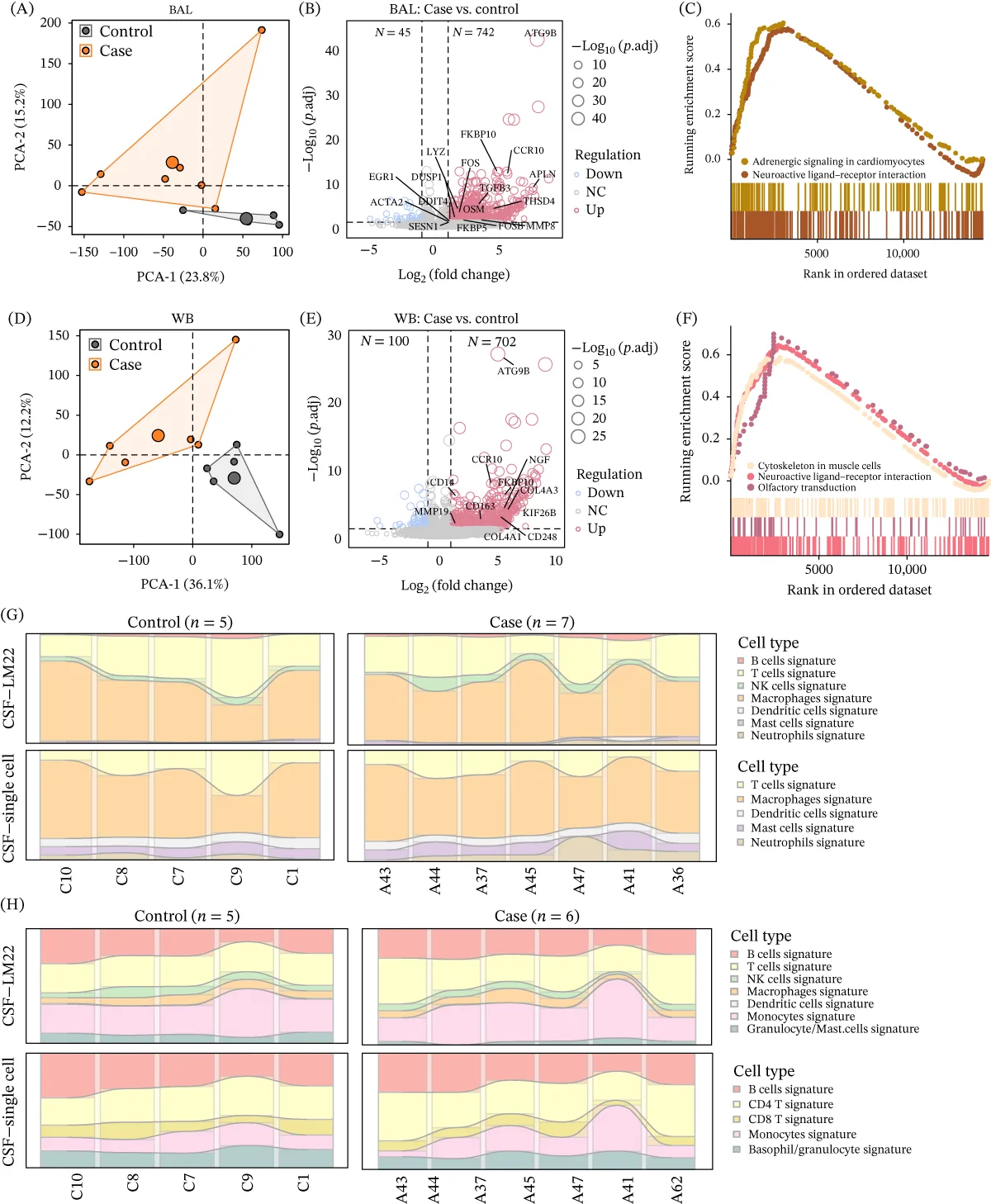
Bulk RNA‐seq analysis of BAL and whole‐blood transcriptomes. (A) PCA analysis of the BAL transcriptome landscape. (B) BAL differential gene expression profile. Volcano plot illustrating 742 upregulated and 45 downregulated genes in equine asthma cases versus controls (*n* = 7 cases, 5 controls, DESeq2). (C) GSEA plots. Two pathways significantly enriched in BAL samples from asthma cases versus controls. (D) PCA analysis of the whole‐blood transcriptome landscape. (E) Whole‐blood differential gene expression profile. Volcano plot illustrating 702 upregulated and 100 downregulated genes in equine asthma cases versus controls (*n* = 6 cases and 5 controls). (F) GSEA plots. Three pathways significantly enriched in whole‐blood samples from asthma cases. (G, H) Cell‐type–associated signatures inferred from bulk RNA‐seq data using CIBERSORTx deconvolution. Stream plots show relative cell‐type–associated signatures across BAL (G) and whole‐blood (H) samples using the LM22 and equine single‐cell–derived reference datasets. These analyses are presented to assess relative differences between groups rather than absolute cellular composition.

Next, we investigated expression patterns in a subset of EA‐mixed cases and controls in BAL samples and WB, two conventionally sampled biological compartments. BAL (7 cases and 5 controls) (Figure 4A–H). Differential expression analysis identified a heterogeneous transcriptional pattern in both, mainly characterized by cellular stress responses, immune activation, and remodeling‐associated transcripts (Figure 4).

In BAL, the most significantly increased transcript was *ATG9B* (log_2_FC: 7.8, *p*  < 10^−10^), a gene involved in autophagosome biogenesis (ref). Together with the upregulation of the stress‐response genes *DDIT4*, *SESN1*, and *FKBP5* (log_2_FC: >1, *p*  < 0.05), this pattern suggested activation of autophagy‐ and cellular stress‐associated programs [42, 43]. Notably, the upregulated genes also included several immune‐ and inflammation‐associated transcripts, including *CCR10*, *MMP8*, *LYZ*, *CLEC4E*, *MS4A7*, and *OSM*, together with immediate‐early response genes, such as *FOS*, *FOSB*, *DUSP1*, and *EGR1*. In parallel, transcripts associated with tissue repair and remodeling, including *APLN*, *FKBP10*, *THSD4*, *TGFB3*, *LOXL3*, and *ACTA2*, were also increased, consistent with structural changes associated with inflammatory airway disease.

Only a very limited number of KEGG pathways were significantly upregulated, possibly as a consequence of low sample numbers and mixed phenotypes. While no canonical immune or inflammatory pathways were identified, enrichment of the cytoskeleton in muscle cells pathway is consistent with alterations in structural and remodeling‐associated processes.

Separate DGE analyses of neutrophilic (*n* = 3) and mastocytic (*n* = 4) cases, although limited by low statistical power, are shown in Supporting Information 14: Figure S5.

WB (6 cases and 5 controls) also exhibited widespread transcriptional alterations, although these showed limited convergence into coherent immune‐associated transcriptional programs (Figure 4D). Among the most significantly increased annotated transcripts were *FKBP10*, *COL4A3*, *CD248*, *KIF26B*, and *NGF*, genes associated with ECM organization, tissue repair, and structural remodeling. *ATG9B*, involved in autophagosome biogenesis, and *CCR10*, a chemokine receptor involved in lymphocyte trafficking, were likewise among the most significantly increased transcripts (Figure 4E). As observed in BAL, only a limited number of KEGG pathways reached significance, none corresponding to canonical inflammatory or asthma‐associated pathways (Figure 4E).

In contrast to the cfRNA findings, which demonstrated marked shifts in B‐cell‐ and T‐cell‐associated signatures, DGE analysis of BAL and WB did not indicate major alterations in lymphocyte‐‐associated transcripts between EA cases and controls. To further investigate potential differences in cellular contribution across cases and controls, we performed CIBERSORTx deconvolution of the BAL and WB bulk RNA‐seq data using both the human LM22 reference matrix [24] and equine BAL [19] and equine PBMC reference [27] datasets derived from single‐cell RNA sequencing. In BAL, inferred cell proportions were concordant between reference matrices (monocyte/macrophages: *r* = 0.897, *p* = 7 × 10^−5^, neutrophils: *ρ* = 0.824, *p* = 0.001, T‐cells: *r* = 0.910, *p* = 0.0004). Similar agreement was observed in WB, where all corresponding correlations exceeded 0.8 (B cells: *r* = 0.910, *p* = 0.0001, monocytes: *ρ* = 0.809, *p* = 0.0026; T cell: *ρ* = 0.927, *p* = 0.00004).

No significant differences in inferred lymphocyte or monocyte/macrophage cell fractions were observed between EA cases and controls using either reference (Figure 4G,H and Supporting Information 13: Table S6). As expected, deconvoluted neutrophil fractions were increased in neutrophilic EA horses compared with controls (LM22: *p* = 0.03577, single cell: *p* = 0.03571). In contrast, inferred mast cell proportions were not increased in mastocytic EA horses and did thus not correlate well with cytological mast cell counts (Supporting Information 15: Figure S6), a pattern consistent with previous observations from equine single‐cell RNA‐seq and flow‐cytometric studies of mast cells in BAL [19, 44].

### 3.5. Comparative Transcriptomic Analysis Across Biological Compartments Identifies Distinct and Overlapping Signatures in EA

**Figure 5:**
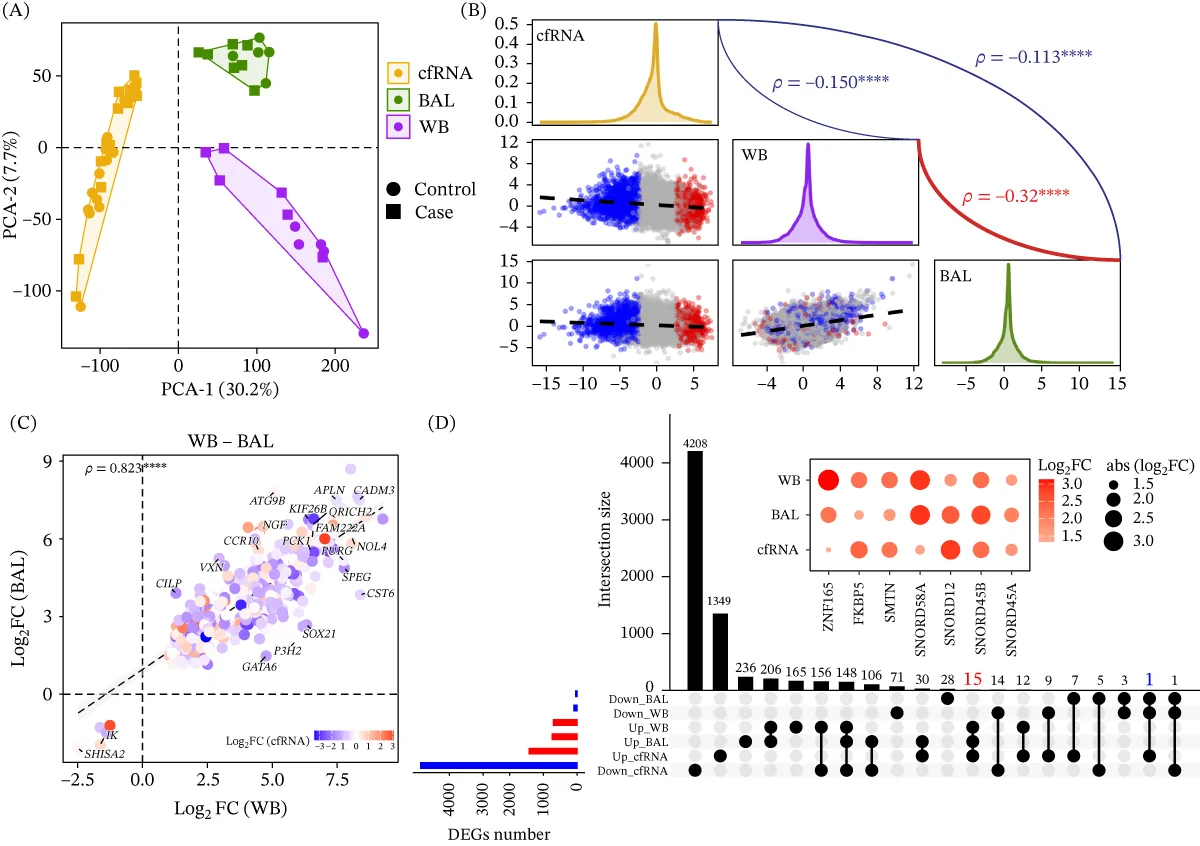
Cross‐compartment analysis of molecular signatures in cfRNA, BAL and whole blood. (A) PCA of TPM values demonstrates a clear separation across the three compartments along PC1 (30.2%) and PC2 (7.7%). (B) Inter‐compartment correlation of differential expression patterns. Pairwise scatter plots display DESeq2 stat value between compartments. Each point represents a gene, colored by its log_2_FC in cfRNA. Density plots show the stat value distributions within each compartment. Correlation coefficients between compartments are indicated in the upper‐right corners. (C) Correlation of log_2_FC for shared DEGs between WB and BAL. Scatter plot showing genes that 374 exhibited concordant up‐ or downregulation in both WB and BAL samples when comparing disease and control groups. The color of each point indicates the corresponding gene’s log_2_ fold change in cfRNA. (D) Comprehensive intersection analysis of differential expression and cross‐compartment biomarker expression profiles. UpSet plot quantifying the overlap patterns of DEGs across all three sample types and regulation directions. Dot plot displaying log_2_FC for seven genes consistently differentially expressed across all three biological compartments.  ^∗∗∗∗^ *p* < 0.0001.

We next sought to further compare transcriptional profiles across the three biological compartments to assess compartment‐specific and overlapping disease signatures in EA. PCA revealed distinct transcriptional landscapes (Figure 5A). Cross‐compartment correlation analysis of differential expression statistics showed weak associations between compartments (Figure 5B). cfRNA exhibited minimal negative correlation with both BAL (*ρ* = −0.113, *p*  < 0.0001) and WB (*ρ* = −0.150, *p*  < 0.0001), while BAL and WB showed modest positive correlation (*ρ* = 0.32, *p*  < 0.0001). Consistent with the modest positive correlation observed between BAL and WB at the global level, 374 genes (369 upregulated and 5 downregulated) were significantly differentially expressed in both compartments. These overlapping DEGs exhibited highly concordant changes in both direction and magnitude (*ρ* = 0.823, *p*  < 0.0001), indicating coordinated transcriptional responses between the airway and peripheral blood (Figure 5C). However, these genes did not show similar expression patterns in cfRNA (Figure 5C).

Among the 369 genes upregulated in both BAL and WB, only 15 were also significantly upregulated in cfRNA, of which eight were annotated as novel genes with an unknown function. Seven annotated transcripts were consistently upregulated across all three compartments, including the structural protein *SMTN*, the stress‐response regulator *FKBP5*, and *ZNF165*, a zinc‐finger transcription factor not previously associated with asthma (Figure 5D). Notably, four small nucleolar RNAs (*SNORD58A*, *SNORD12*, *SNORD45B*, and *SNORD45A*) were consistently more abundant across cfRNA, BAL, and WB, consistent with altered RNA‐processing pathways (Figure 5D). More broadly, large numbers of snoRNAs were differentially expressed in all three compartments (cfRNA: 93, BAL: 77, and WB: 37), indicating widespread alterations in noncoding RNA abundance (Supporting Information 6: Data 3).

Collectively, these analyses suggest that plasma cfRNA captures a molecular signature largely distinct from that observed in BAL and WB, while also revealing a limited set of shared transcripts across biological compartments in this cohort. Among the most notable shared signals were FKBP5, a stress‐response regulator previously implicated in both equine and human asthma [19] and multiple small nucleolar RNA.

## 4. Discussion

In this study, we performed the first characterization of equine plasma cfRNA and compared transcriptional signatures between horses with EA and healthy controls, as well as across biological compartments. The principal finding was that plasma cfRNA captured disease‐associated molecular patterns in EA, including signatures related to epithelial activation, tissue remodeling, cellular stress responses, and immune alterations. Notably, these signals were only partly reflected in BAL and WB transcriptomes, suggesting that plasma cfRNA provides molecular information complementary to conventional sampling approaches. Nevertheless, the cross‐compartment comparisons should be regarded as exploratory, as the smaller and more heterogeneous BAL and WB cohorts may have limited the ability to detect shared disease‐associated molecular patterns. More broadly, our results support the growing concept that plasma liquid biopsies can serve as a source of RNA‐based biomarkers in chronic inflammatory diseases [45].

Among individual transcripts, *IL33* emerged as one of the most strongly increased transcripts in plasma cfRNA from horses with EA. *IL33* is a well‐established alarmin produced by epithelial, endothelial, and stromal cells and implicated in airway inflammation, tissue injury, and remodeling in both allergic and nonallergic respiratory diseases. Increased detection of *IL33* transcripts in plasma may therefore be consistent with epithelial activation or tissue damage occurring within the respiratory tract [46]. The concurrent enrichment of ECM remodeling, platelet–endothelial activation, and cellular stress‐response signatures further supports the presence of ongoing tissue injury and repair processes in EA. However, the precise cellular origin of circulating *IL33* transcripts and other upregulated transcripts cannot be determined from the present data and these findings should therefore be interpreted cautiously. Nonetheless, the prominent detection of *IL33* in circulating cfRNA suggests that it may represent a candidate minimally invasive biomarker of airway epithelial perturbation in EA.

In addition, *IL13RA1*, *RORA*, and *IGHE* were among the transcripts showing increased abundance in plasma cfRNA from EA horses. In humans, these genes have previously been implicated in allergic airway disease and type‐2 immune responses [31, 47]. However, the biological significance of their increased abundance in equine plasma cfRNA remains unclear and warrants further investigation. Interestingly, *FKBP5*, a regulator of glucocorticoid signaling and cellular stress responses that has previously been implicated in both equine and human asthma, was consistently increased across all three biological compartments, suggesting that it may represent a systemic molecular marker of EA [19, 48].

Plasma cfRNA enters the circulation through cellular turnover, cell death, and active secretion within EVs. Deconvolution analyses suggested contributions from diverse cell and tissue compartments, including epithelial, stromal, gastrointestinal, and muscle‐associated cell populations. Consistent with observations from human plasma cfRNA studies, immune cell‐associated signatures were prominently represented within the equine plasma cfRNA profile. This may be particularly relevant in immune‐mediated diseases, as the majority of lymphocytes reside within tissues rather than in the circulation, especially at barrier sites such as the respiratory and gastrointestinal tracts [49]. Consequently, plasma cfRNA may capture signals originating from tissue‐resident immune populations that are not readily detected in peripheral blood transcriptomes. Consistent with this possibility, deconvolution analyses identified reduced B‐cell‐associated and increased T‐cell‐associated signatures in EA plasma cfRNA, whereas comparable immune‐associated alterations were not evident in the BAL or WB transcriptomes. Importantly, these inferred cell‐type‐associated signatures should not be interpreted as direct estimates of changes in immune cell abundance, as circulating cfRNA represents a composite signal influenced by RNA release, tissue contributions, cell turnover, EV dynamics, and RNA clearance. Rather, they are consistent with altered immune‐associated molecular contributions to the circulating cfRNA pool. Importantly, these observations were also supported by differential abundance, pathway, and network analyses. This convergence across multiple analytical approaches increases confidence that the observed patterns reflect underlying biological signals rather than the artifacts of cross‐species reference mapping alone.

This study has several limitations. First, the cohort size was modest, limiting statistical power and preventing robust phenotype‐specific analyses. Second, cfRNA, BAL, and WB samples were only partially matched across individuals, which may have reduced the apparent overlap between compartments. Third, EA cases represented a mixture of clinical phenotypes that may differ biologically. Fourth, the control group consisted of a single breed, whereas the EA cases comprised multiple breeds. Fifth, cell and tissue deconvolution for plasma cfRNA relied primarily on cross‐species reference datasets, introducing potential annotation bias and uncertainty in inferred cellular contributions.

Despite these limitations, our data provide an initial framework for understanding the circulating transcriptome in EA and highlight the potential of plasma cfRNA as a source of molecular information in naturally occurring inflammatory airway diseases. Future studies should validate these findings in larger and longitudinal cohorts, integrate cfRNA profiles with clinical and physiological measures, and leverage emerging equine pan‐tissue single‐cell reference atlases to improve tissue‐of‐origin analyses.

**Nomenclature**
cfRNA: Cell‐free RNA
EA: Equine asthma
BAL: Bronchoalveolar lavage
sEA: Severe EA
mEA: Mild‐to‐moderate EA
WB: Whole blood
ORA: Over‐representation analysis
GSEA: Gene set enrichment analysis
CPM: counts per million
WGCNA: Weighted gene co‐expression network analysis
DEGs: Differentially expressed genes
VST: variance‐stabilizing transformation
PCA: Principal component analysis
TPM: Transcripts per million
ECM: Extracellular matrix
NES: Normalized enrichment score
MEs: Modules.

## Author Contributions

Birong Zhang analyzed the data, interpreted results, and wrote the manuscript together with Amanda Raine. Hanna Agdour assisted in sampling, performed the lab work, and interpretation of results. Miia Riihimäki performed sampling of clinical cases and clinical assessments. Miia Riihimäki and Sanni Hansen performed sampling of controls. Miia Riihimäki and Sanni Hansen provided expertise in equine asthma. Amanda Raine conceived and supervised the study, interpreted results, acquired funding, and drafted the manuscript.

## Acknowledgments

The sequencing data were generated with assistance from the SciLifeLab National Genomics Infrastructure, SNP&SEQ Technology Platform, which is funded by the Swedish Research Council and the Knut and Alice Wallenberg Foundation. The raw bulk RNA‐Seq analysis process was performed on resources provided by the Uppsala Multidisciplinary Center for Advanced Computational Science (UPPMAX) at Uppsala University and the National Academic Infrastructure for Supercomputing in Sweden (NAISS). We thank all horse owners for participating in the study and the staff at Wången for their assistance.

## Funding

This work was mainly supported by a grant from The Swedish‐Norwegian Foundation for Equine Research (Grant H‐22‐47‐717) and in part by the FORMAS (Grant 2023‐01377). Open‐access funding was provided by the Uppsala University.

## Disclosure

All authors have read and edited the manuscript. A preprint (https://www.biorxiv.org/content/10.1101/2025.10.25.684590v1) [50] of the study has previously been published.

## Ethics Statement

All animal procedures were approved by the Regional Ethical Committee (Permits #5.2.18‐4819/15 and #31‐2605/04) and conducted in accordance with the relevant guidelines and regulations. Horse owners provided written informed consent.

## Consent

The authors have nothing to report.

## Conflicts of Interest

The authors declare no conflicts of interest.

## Supporting Information

Additional supporting information can be found online in the Supporting Information section.

## Supporting information


**Supporting Information 1** Table S1: BAL cytology and phenotypes.

**Supporting Information 2** Table S2: Subject demographics by disease status and sample type.

**Supporting Information 3** Data 1: Read alignment metrics.

**Supporting Information 4** Data 2: Summary of statistical tests used for all figures.

**Supporting Information 5** Table S3: Gene biotype composition.

**Supporting Information 6** Data 3: Differentially expressed genes.

**Supporting Information 7** Table S4: Kegg pathway analysis case vs control cfRNA.

**Supporting Information 8** Figure S1: cfRNA WGCNA profiling. (A, B) Weighted gene co‐expression network analysis (WGCNA). The two most significant co‐expressed modules: module_blue (upregulated) and module_turquoise (downregulated). Correlation plots between gene significance and module membership. (C) Heatmap of genes in module_blue and module_turquoise. Potential asthma‐related genes (identified from human literature) are highlighted on the left, with corresponding over‐representation analysis results shown on the right.

**Supporting Information 9** Table S5: WGCNA gene info.

**Supporting Information 10** Figure S2: cfRNA profiling based on BAL cytological phenotypes. (A) PCA of cfRNA gene expression profiles showing separation among control (gray), mastocytic (pink), and neutrophilic (green) phenotypes along PC1 (14.8%) and PC2 (9.0%). (B) Differential gene expression profile comparing mastocytic and control cfRNA samples. (C) Differential gene expression profile comparing neutrophilic and control cfRNA samples. (D) UpSet plot showing overlap of DEGs between mastocytic vs. control and neutrophilic vs. control comparisons. (E) Correlation of log_2_FC values for shared DEGs across the two BAL cytological phenotype comparisons. (F) UpSet plot showing overlap of enriched pathways across the two BAL cytological phenotype comparisons. (G) 10/65 shared pathways identified across mastocytic and neutrophilic analyses.

**Supporting Information 11** Figure S3: cfRNA CibersortX cell deconvolution profiling. Box plots depict relative proportions of 22 distinct immune cell populations estimated using LM22‐based deconvolution across control (gray, *n* = 12) and asthmatic case (orange, *n* = 16) samples derived from cfRNA sampling. Each panel corresponds to a specific immune cell subset, with cell signature fraction on the *y*‐axis. Boxplot shows group medians with interquartile range (IQRs; 25th–75th percentiles).

**Supporting Information 12** Figure S4: Cross‐method validation of B‐cell and T‐cell signature alterations identified by CIBERSORTx. Scatter plots compare cell fractions estimated by CIBERSORTx (*x*‐axis) against two independent deconvolution methods (*y*‐axis). Correlation coefficients and *p*‐values are shown for scatter plots, and Wilcoxon *p*‐values for boxplots. Each dot represents an individual cfRNA sample, colored by group (gray, control: *n* = 12; orange, case: *n* = 16). (A) wls with the TSP reference. (B) dtangle with the TSP single‐cell reference. (C) wls with the LM22 reference. (D) dtangle with the LM22 reference. (E) xCell estimates shown as boxplots comparing control and case groups.

**Supporting Information 13** Table S6: Comparative cell type statistics.

**Supporting Information 14** Figure S5: BAL transcriptomic profiling based on cytological phenotypes. (A) PCA of BAL gene expression profiles showing clear separation among control (gray), mastocytic (pink), and neutrophilic (green) phenotypes along PC1 (23.8%) and PC2 (15.2%). (B) Differential gene expression profile comparing mastocytic and control BAL samples. (C) Differential gene expression profile comparing neutrophilic and control BAL samples. (D) Pathway‐level differences between mastocytic and control groups. (E) Pathway‐level differences between neutrophilic and control groups..

**Supporting Information 15** Figure S6: Comparison of bulk BAL RNA‐seq deconvolution estimates with BAL cytology fractions. (A, B) Scatter plots showing correlations between cell fractions estimated by deconvolution of bulk BAL RNA‐seq data (*y*‐axis) and corresponding cytological fractions (*x*‐axis) for four cell types. Each point represents an individual BAL sample and is colored according to cytological phenotype (gray, control: *n* = 5; pink, mastocytic: *n* = 4; green, neutrophilic: *n* = 3). (A) LM22 reference matrix‐based deconvolution. (B) Equine scRNA‐seq‐derived reference matrix‐based deconvolution.

## Data Availability

The data that support the findings of this study are openly available in the NCBI SRA at https://www.ncbi.nlm.nih.gov/bioproject/?term=PRJNA1354409. The code for analysis and the figure in this study is found in the github repository https://github.com/Molmed/Equine_cfRNA.
