# Supplementary material for: Circulating Cell‐Free RNA Reflects Immune‐Associated and Airway Remodeling Signatures in Equine Asthma
Source: J Immunol Res. 2026 Aug 26;2026:5310284. doi: 10.1155/jimr/5310284 (PMC13509420; doi:10.1155/jimr/5310284)
Supplement: Supplementary file 1 [file JIMR-2026-5310284-s007.pdf]

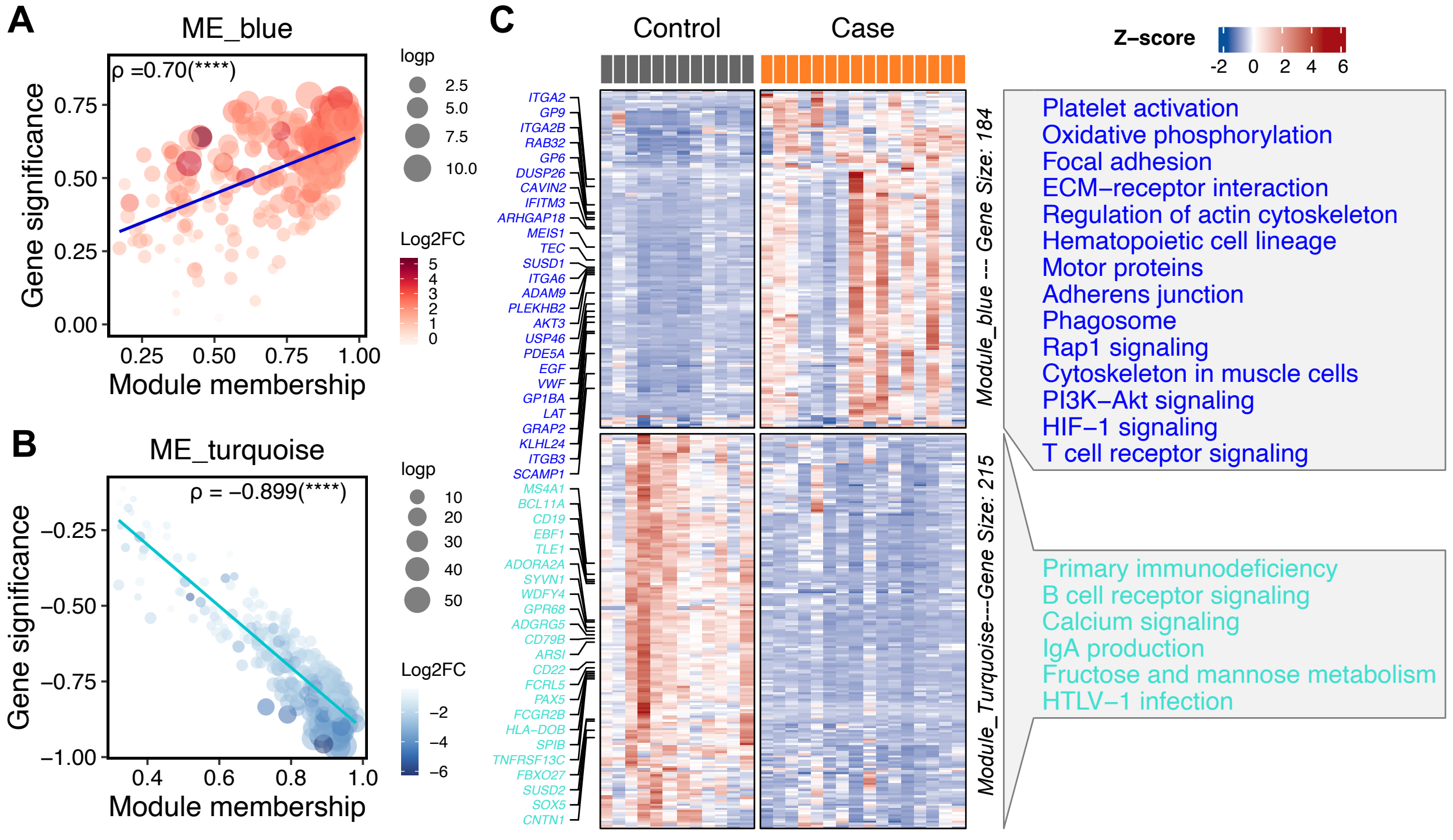

A
ME_blue
ρ =0.70(****)
Gene significance
Module membership
0.00
0.25
0.50
0.75
1.00
logp
2.5
5.0
7.5
10.0
Log2FC
5
4
3
2
1
0
B
ME_turquoise
ρ = −0.899(****)
Gene significance
Module membership
−0.25
−0.50
−0.75
−1.00
0.4
0.6
0.8
1.0
logp
10
20
30
40
50
Log2FC
−2
−4
−6
C
Control
Case
Z−score
-2
0
2
4
6
ITGA2
GP9
ITGA2B
RAB32
GP6
DUSP26
CAVIN2
IFITM3
ARHGAP18
MEIS1
TEC
SUSD1
ITGA6
ADAM9
PLEKHB2
AKT3
USP46
PDE5A
EGF
VWF
GP1BA
LAT
GRAP2
KLHL24
ITGB3
SCAMP1
MS4A1
BCL11A
CD19
EBF1
TLE1
ADORA2A
SYVN1
WDFY4
GPR68
ADGRG5
CD79B
ARSI
CD22
FCRL5
PAX5
FCGR2B
HLA−DOB
SPIB
TNFRSF13C
FBXO27
SUSD2
SOX5
CNTN1
Module_blue --- Gene Size: 184
Module_Turquoise---Gene Size: 215
Platelet activation
Oxidative phosphorylation
Focal adhesion
ECM−receptor interaction
Regulation of actin cytoskeleton
Hematopoietic cell lineage
Motor proteins
Adherens junction
Phagosome
Rap1 signaling
Cytoskeleton in muscle cells
PI3K−Akt signaling
HIF−1 signaling
T cell receptor signaling
Primary immunodeficiency
B cell receptor signaling
Calcium signaling
IgA production
Fructose and mannose metabolism
HTLV−1 infection
