# Supplementary material for: Circulating Cell‐Free RNA Reflects Immune‐Associated and Airway Remodeling Signatures in Equine Asthma
Source: J Immunol Res. 2026 Aug 26;2026:5310284. doi: 10.1155/jimr/5310284 (PMC13509420; doi:10.1155/jimr/5310284)
Supplement: Supplementary file 2 [file JIMR-2026-5310284-s009.pdf]

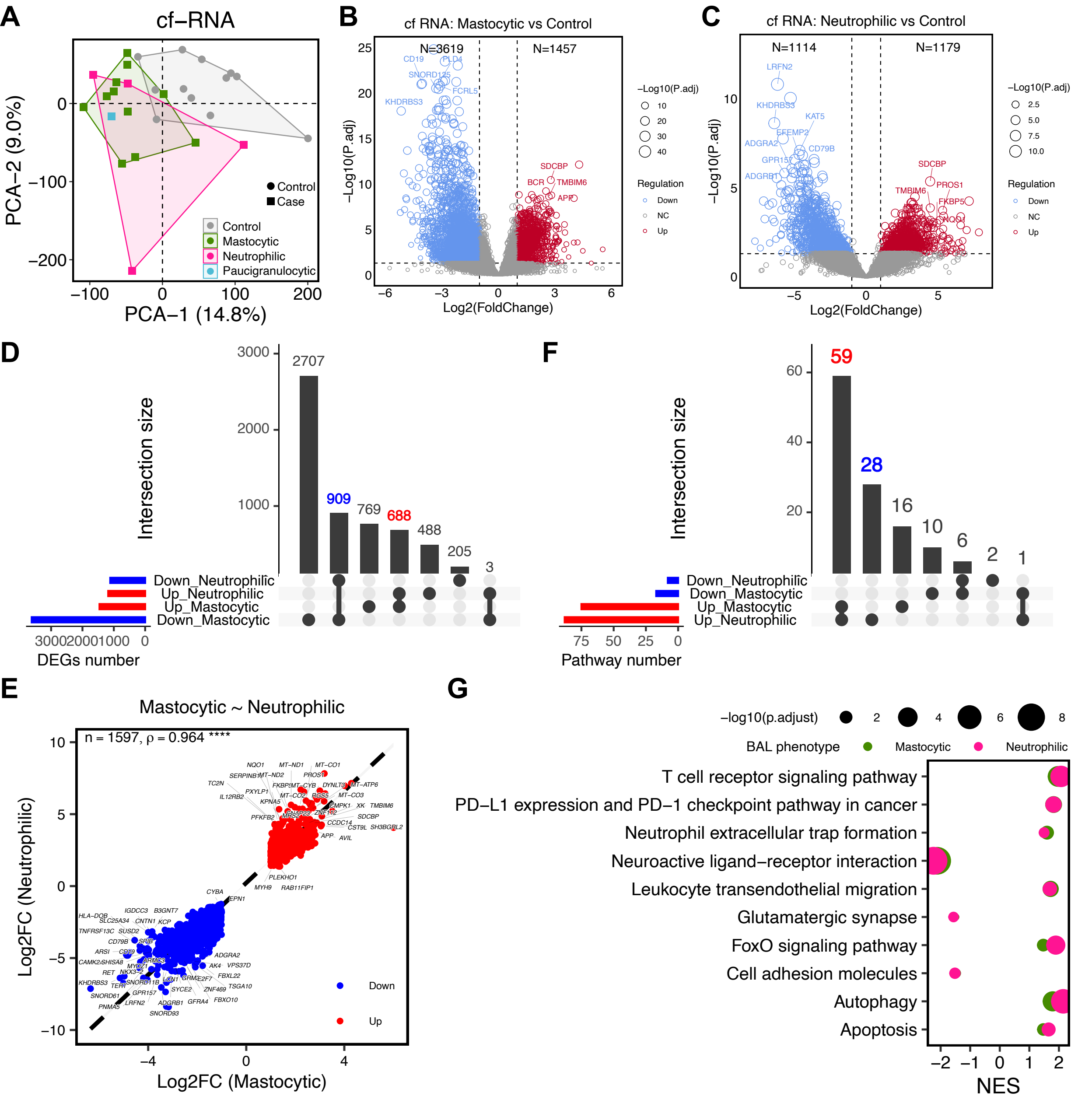
A
cf−RNA
PCA−1 (14.8%)
PCA−2 (9.0%)
Control
Case
Control
Mastocytic
Neutrophilic
Paucigranulocytic
B
cf RNA: Mastocytic vs Control
N=3619
N=1457
CD19
PLD4
SNORD125
FCRL5
KHDRBS3
SDCBP
BCR
TMBIM6
APP
Log2(FoldChange)
−Log10(P.adj)
−Log10(P.adj)
Regulation
Down
NC
Up
C
cf RNA: Neutrophilic vs Control
N=1114
N=1179
LRFN2
KHDRBS3
KAT5
EFEMP2
ADGRA2
CD79B
GPR157
ADGRB1
SDCBP
TMBIM6
PROS1
FKBP5
Log2(FoldChange)
D
Intersection size
2707
909
769
688
488
205
3
Down_Neutrophilic
Up_Neutrophilic
Up_Mastocytic
Down_Mastocytic
DEGs number
F
Intersection size
59
28
16
10
6
2
1
Down_Neutrophilic
Down_Mastocytic
Up_Mastocytic
Up_Neutrophilic
Pathway number
E
Mastocytic ~ Neutrophilic
n = 1597, ρ = 0.964 ****
Log2FC (Mastocytic)
Log2FC (Neutrophilic)
Down
Up
G
−log10(p.adjust)
BAL phenotype
Mastocytic
Neutrophilic
T cell receptor signaling pathway
PD−L1 expression and PD−1 checkpoint pathway in cancer
Neutrophil extracellular trap formation
Neuroactive ligand−receptor interaction
Leukocyte transendothelial migration
Glutamatergic synapse
FoxO signaling pathway
Cell adhesion molecules
Autophagy
Apoptosis
NES
