# Supplementary material for: Circulating Cell‐Free RNA Reflects Immune‐Associated and Airway Remodeling Signatures in Equine Asthma
Source: J Immunol Res. 2026 Aug 26;2026:5310284. doi: 10.1155/jimr/5310284 (PMC13509420; doi:10.1155/jimr/5310284)
Supplement: Supplementary file 3 [file JIMR-2026-5310284-s010.pdf]

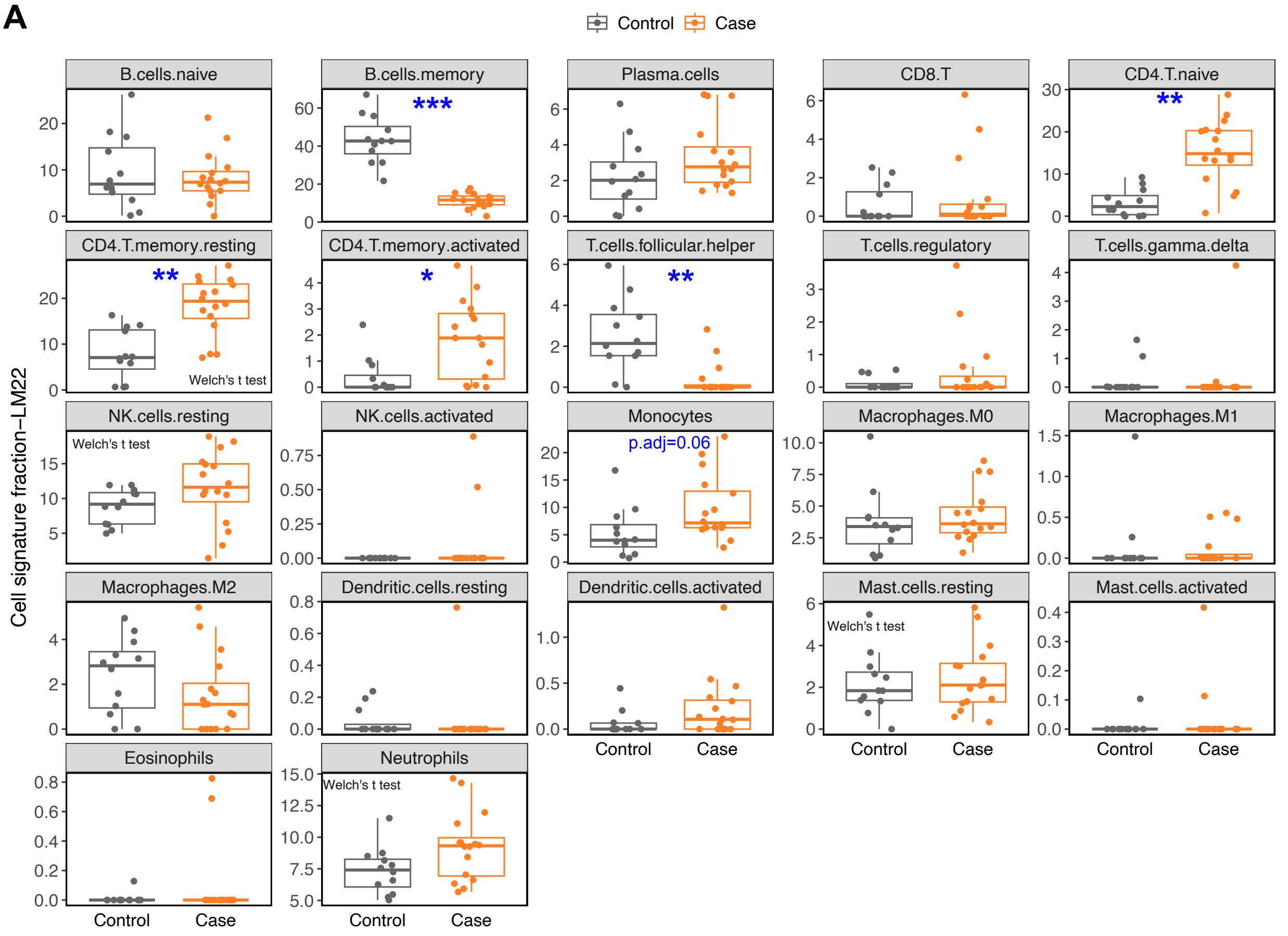

A
Control
Case
Cell signature fraction−LM22
B.cells.naive
B.cells.memory
***
Plasma.cells
CD8.T
CD4.T.naive
**
CD4.T.memory.resting
**
Welch's t test
CD4.T.memory.activated
*
T.cells.follicular.helper
**
T.cells.regulatory
T.cells.gamma.delta
NK.cells.resting
Welch's t test
NK.cells.activated
Monocytes
p.adj=0.06
Macrophages.M0
Macrophages.M1
Macrophages.M2
Dendritic.cells.resting
Dendritic.cells.activated
Mast.cells.resting
Welch's t test
Mast.cells.activated
Eosinophils
Neutrophils
Welch's t test
