# Supplementary material for: Circulating Cell‐Free RNA Reflects Immune‐Associated and Airway Remodeling Signatures in Equine Asthma
Source: J Immunol Res. 2026 Aug 26;2026:5310284. doi: 10.1155/jimr/5310284 (PMC13509420; doi:10.1155/jimr/5310284)
Supplement: Supplementary file 4 [file JIMR-2026-5310284-s011.pdf]

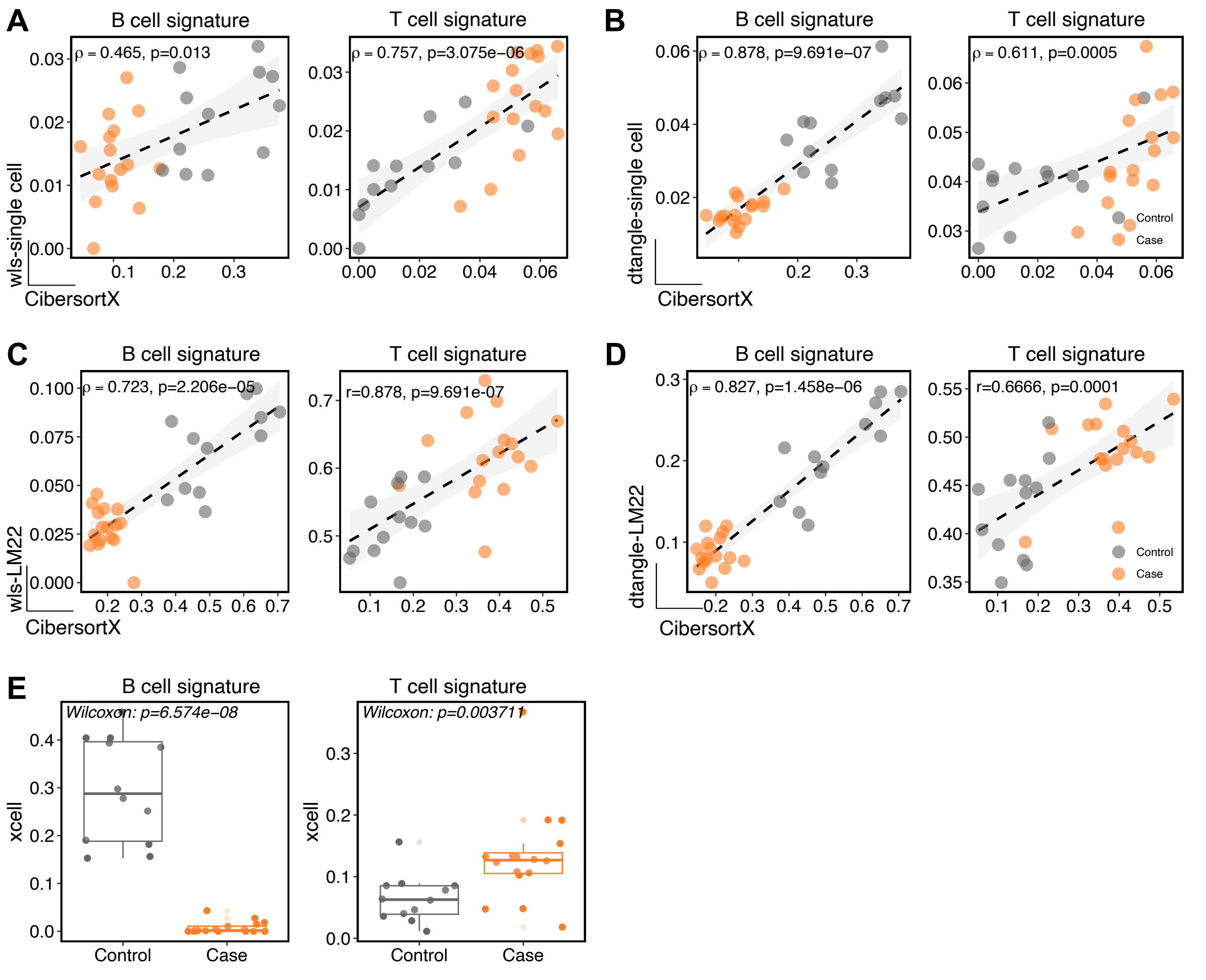
A
B cell signature
ρ = 0.465, p=0.013
T cell signature
ρ = 0.757, p=3.075e−06
wls-single cell
CibersortX
B
B cell signature
ρ = 0.878, p=9.691e−07
T cell signature
ρ = 0.611, p=0.0005
dtangle-single cell
CibersortX
Control
Case
C
B cell signature
ρ = 0.723, p=2.206e−05
T cell signature
r=0.878, p=9.691e-07
wls-LM22
CibersortX
D
B cell signature
ρ = 0.827, p=1.458e−06
T cell signature
r=0.6666, p=0.0001
dtangle-LM22
CibersortX
Control
Case
E
B cell signature
Wilcoxon: p=6.574e−08
xcell
Control
Case
T cell signature
Wilcoxon: p=0.003711
xcell
Control
Case
