# Supplementary material for: Circulating Cell‐Free RNA Reflects Immune‐Associated and Airway Remodeling Signatures in Equine Asthma
Source: J Immunol Res. 2026 Aug 26;2026:5310284. doi: 10.1155/jimr/5310284 (PMC13509420; doi:10.1155/jimr/5310284)
Supplement: Supplementary file 5 [file JIMR-2026-5310284-s013.pdf]

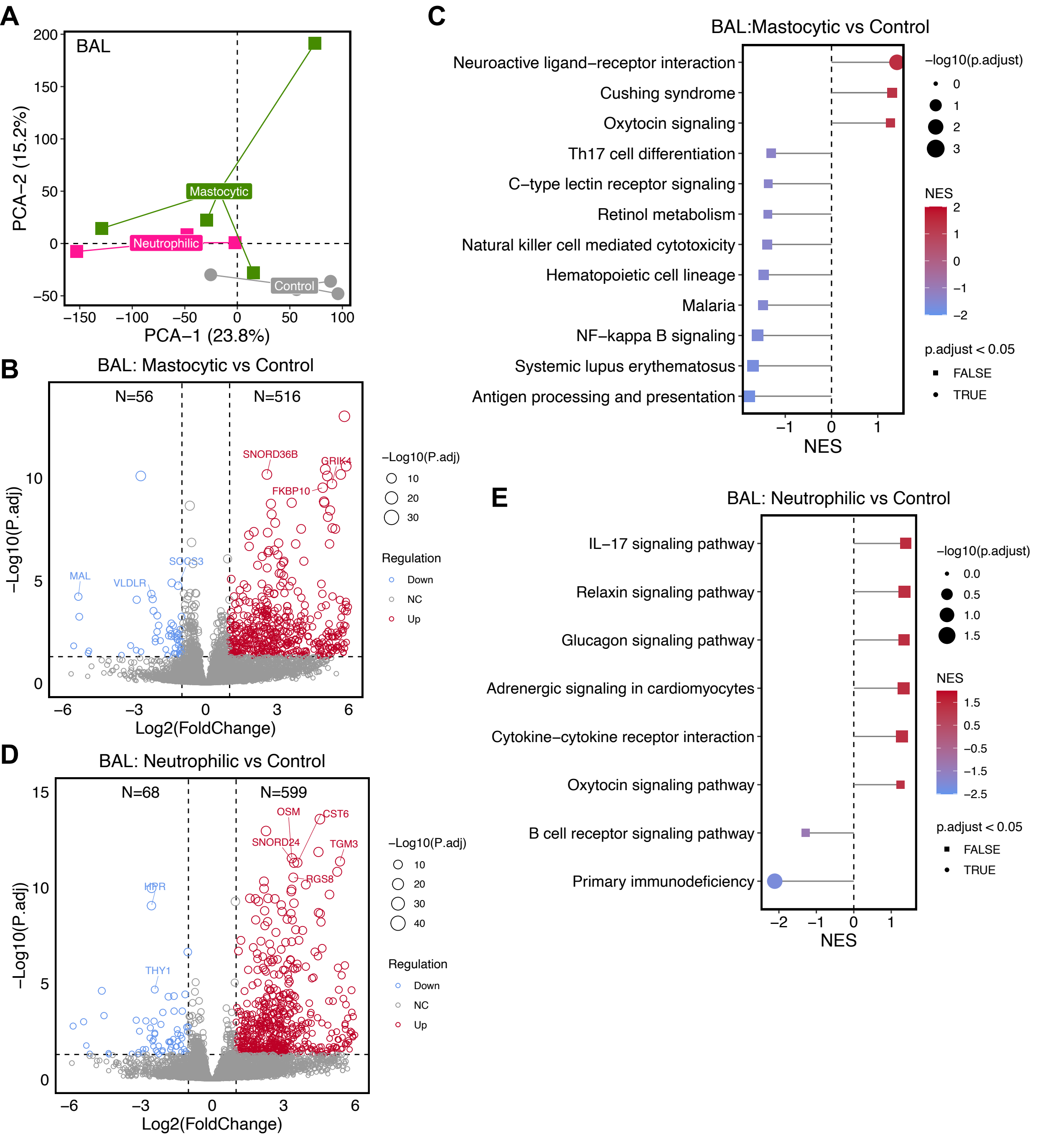

A
BAL
PCA−2 (15.2%)
PCA−1 (23.8%)
Mastocytic
Neutrophilic
Control
B
BAL: Mastocytic vs Control
N=56
N=516
SNORD36B
GRIK4
FKBP10
SOCS3
MAL
VLDLR
−Log10(P.adj)
Log2(FoldChange)
−Log10(P.adj)
10
20
30
Regulation
Down
NC
Up
C
BAL:Mastocytic vs Control
Neuroactive ligand−receptor interaction
Cushing syndrome
Oxytocin signaling
Th17 cell differentiation
C−type lectin receptor signaling
Retinol metabolism
Natural killer cell mediated cytotoxicity
Hematopoietic cell lineage
Malaria
NF−kappa B signaling
Systemic lupus erythematosus
Antigen processing and presentation
NES
−log10(p.adjust)
NES
p.adjust < 0.05
FALSE
TRUE
D
BAL: Neutrophilic vs Control
N=68
N=599
OSM
CST6
SNORD24
TGM3
RGS8
HPR
THY1
−Log10(P.adj)
Log2(FoldChange)
−Log10(P.adj)
10
20
30
40
Regulation
Down
NC
Up
E
BAL: Neutrophilic vs Control
IL−17 signaling pathway
Relaxin signaling pathway
Glucagon signaling pathway
Adrenergic signaling in cardiomyocytes
Cytokine−cytokine receptor interaction
Oxytocin signaling pathway
B cell receptor signaling pathway
Primary immunodeficiency
NES
−log10(p.adjust)
NES
p.adjust < 0.05
FALSE
TRUE
