# Supplementary material for: Circulating Cell‐Free RNA Reflects Immune‐Associated and Airway Remodeling Signatures in Equine Asthma
Source: J Immunol Res. 2026 Aug 26;2026:5310284. doi: 10.1155/jimr/5310284 (PMC13509420; doi:10.1155/jimr/5310284)
Supplement: Supplementary file 6 [file JIMR-2026-5310284-s014.pdf]

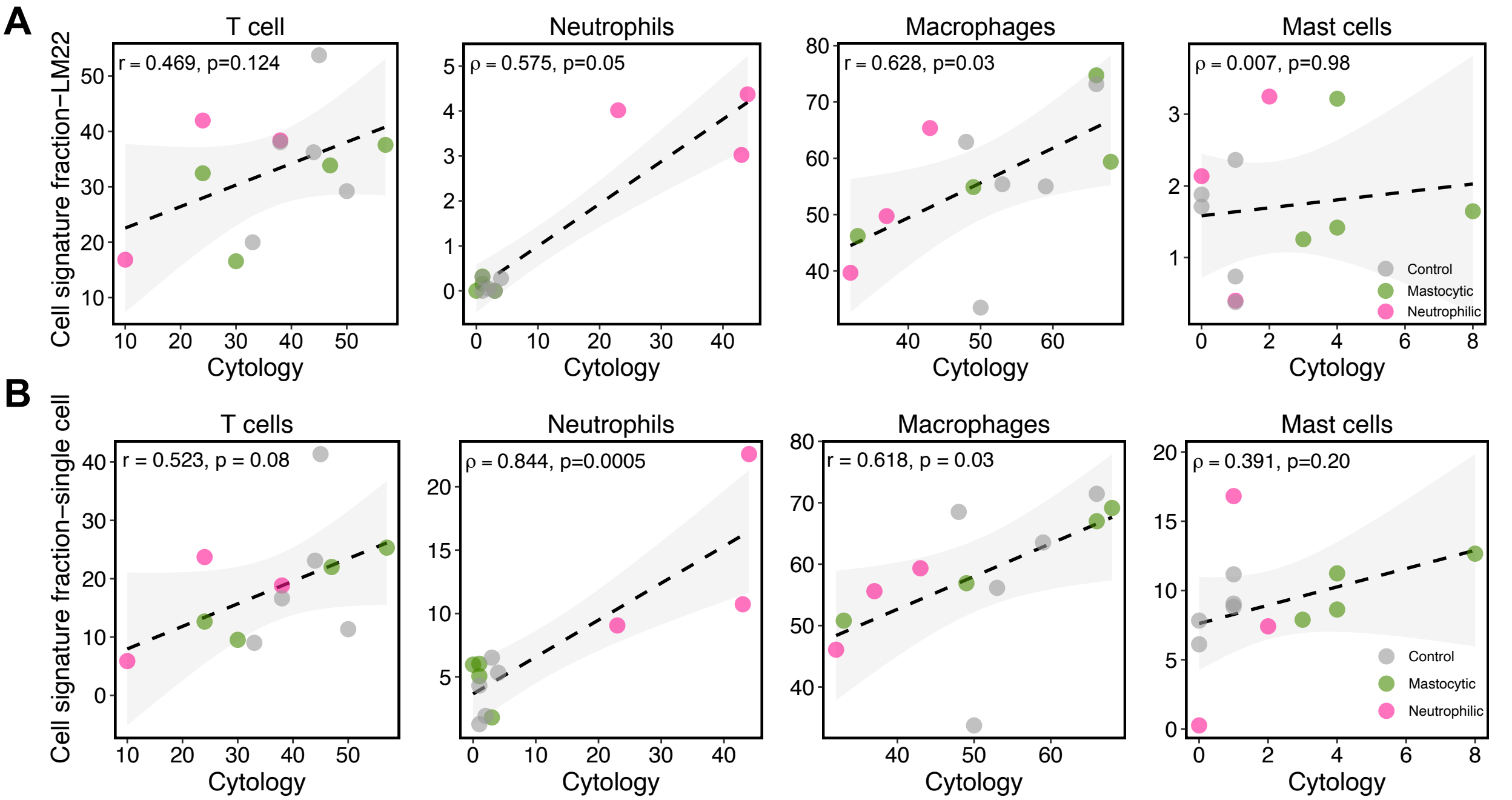

A
Cell signature fraction−LM22
T cell
r = 0.469, p=0.124
Neutrophils
ρ = 0.575, p=0.05
Macrophages
r = 0.628, p=0.03
Mast cells
ρ = 0.007, p=0.98
Control
Mastocytic
Neutrophilic
Cytology
B
Cell signature fraction−single cell
T cells
r = 0.523, p = 0.08
Neutrophils
ρ = 0.844, p=0.0005
Macrophages
r = 0.618, p = 0.03
Mast cells
ρ = 0.391, p=0.20
Control
Mastocytic
Neutrophilic
Cytology
