# Supplementary material for: Circulating Cell‐Free RNA Reflects Immune‐Associated and Airway Remodeling Signatures in Equine Asthma
Source: J Immunol Res. 2026 Aug 26;2026:5310284. doi: 10.1155/jimr/5310284 (PMC13509420; doi:10.1155/jimr/5310284)
Supplement: Supplementary file 7 [file JIMR-2026-5310284-s001.docx]

|  | | **cf-RNA** | | | **BAL cells** | | | **Whole blood** | | |
| --- | --- | --- | --- | --- | --- | --- | --- | --- | --- | --- |
| **Characteristic** | | **Control**  N = 12*^1^* | **Case**  N = 16*^1^* | **p-value***^2^* | **Control**  N = 5*^1^* | **Case**  N = 7*^1^* | **p-value***^2^* | **Control**  N = 5*^1^* | **Case**  N = 6*^1^* | **p-value***^2^* |
| **Age** | | 9 (3) | 13 (5) | 0.006 | 8 (3) | 15 (3) | 0.002 | 8 (3) | 13 (5) | 0.060 |
| **Sex** | |  |  | >0.9 |  |  | 0.2 |  |  | 0.2 |
| gelding | | 8 (67%) | 11 (69%) |  | 5 (100%) | 4 (57%) |  | 5 (100%) | 3 (50%) |  |
| mare | | 4 (33%) | 5 (31%) |  | 0 (0%) | 3 (43%) |  | 0 (0%) | 3 (50%) |  |
|  | *^1^*Mean (SD); n (%) | | | | | | | | | |
|  | *^2^*Welch’s t-test(age); Fisher's exact test (sex) | | | | | | | | | |

**Supplementary Table 2. Subject demographics by disease status and sample type.**
